# A Disposable Organophosphorus Pesticides Enzyme Biosensor Based on Magnetic Composite Nano-Particles Modified Screen Printed Carbon Electrode

**DOI:** 10.3390/s100100625

**Published:** 2010-01-15

**Authors:** Ning Gan, Xin Yang, Donghua Xie, Yuanzhao Wu, Weigang Wen

**Affiliations:** 1 The State Key Laboratory Base of Novel Functional Materials and Preparation Science, Faculty of Material Science and Chemical Engineering of Ningbo University, Ningbo, 315211, China; E-Mails: 01yangxin@163.com (X.Y.); wyznanhang@163.com (Y.Z.W.); 2 The Technical Center of Ningbo Entry-Exit Inspection and Quarantine Bureau, Ningbo, 315200, China; E-Mail: xiedh@nbciq.gov.cn (D.H.X.)

**Keywords:** Fe_3_O_4_/Au composite nanoparticles, carbon nanotubes, nano-ZrO_2_, acetyl-cholinesterase, organophosphorus pesticides, prussian blue, screen-printed carbon electrodes, enzyme biosensor

## Abstract

A disposable organophosphorus pesticides (OPs) enzyme biosensor based on magnetic composite nanoparticle-modified screen printed carbon electrodes (SPCE) has been developed. Firstly, an acetylcholinesterase (AChE)-coated Fe_3_O_4_/Au (GMP) magnetic nanoparticulate (GMP-AChE) was synthesized. Then, GMP-AChE was absorbed on the surface of a SPCE modified by carbon nanotubes (CNTs)/nano-ZrO_2_/prussian blue (PB)/Nafion (Nf) composite membrane by an external magnetic field. Thus, the biosensor (SPCE│CNTs/ZrO_2_/PB/Nf│GMP-AChE) for OPs was fabricated. The surface of the biosensor was characterized by scanning electron micrography (SEM) and X-ray fluorescence spectrometery (XRFS) and its electrochemical properties were studied by cyclic voltammetry (CV) and differential pulse voltammetry (DPV). The degree of inhibition (*A*%) of the AChE by OPs was determined by measuring the reduction current of the PB generated by the AChE-catalyzed hydrolysis of acetylthiocholine (ATCh). In pH = 7.5 KNO_3_ solution, the *A* was related linearly to the concentration of dimethoate in the range from 1.0 × 10^−3^–10 ng·mL^−1^ with a detection limit of 5.6 × 10^−4^ ng·mL^−1^. The recovery rates in Chinese cabbage exhibited a range of 88%–105%. The results were consistent with the standard gas chromatography (GC) method. Compared with other enzyme biosensors the proposed biosensor exhibited high sensitivity, good selectivity with disposable, low consumption of sample. In particular its surface can be easily renewed by removal of the magnet. The convenient, fast and sensitive voltammetric measurement opens new opportunities for OPs analysis.

## 1. Introduction

Organophosphorus pesticides (OPs) are widely used in agriculture due to their high toxicity to insects and limited persistence in the environment, but this use has resulted in a serious risk to human health and the environment worldwide. Many efforts have been made to develop sensitive, convenient and effective methods for OP residues analysis in environmental and other samples [1,2]. Gas chromatography (GC) [3,4], GC-Mass Spectrometry (MS) [5], and high performance liquid chromatography (HPLC)-MS [6–8] are commonly used methods to successfully detect OP residues in practical samples with high selectivity, sufficient sensitivity and precision. However, these methods require highly skilled personnel, specially equipped laboratories, and expensive chemicals which limit their extensive application in screening determination of trace OP residues [9–11]. Therefore, it is highly desirable to develop new methods to screen and conveniently monitor OPs.

At present, the combination of enzymatic reactions (inhibition of acetylcholinesterase, AChE) with amperometric electrochemical methods has enabled the development of a variety of enzyme-based electrochemical biosensors for sensitive and rapid determination of OPs [8–11]. In particular, enzyme biosensors fabricated on screen printed cabon electrodes (SPCE) have the advantages of integration of electrodes, simple manipulations, low cost and low consumption of sample which can be used for one step determination then discarded [12,13].

Up to now, due to their high surface area, good stability and biocompatibility, more and more attention has been paid to some conductive nanoparticle materials, such as gold colloids, carbon nanotubes (CNTs), Fe_3_O_4_ and so on, to fabricate functional-film modified electrochemical biosensors [14,15]. Among them, CNTs present a singular structure and dimensions together with unique electronic and chemical properties. As an electrode material, CNTs can facilitate electron-transfer between the electroactive species and the electrode. This makes CNTs a very attractive material to develop supersensitive electrochemical biosensors due to their outstanding ability to promote the electron transfer reactions of a great number of redox molecules [16–18]. Au colloid coated Fe_3_O_4_ magnetic nanoparticles (Fe_3_O_4_/Au, gold magnetic particles, abbreviation as GMP) were selected for the immobilization of AChE to fabricate the biosensor. The choice of GMP is based on the magnetic feature of Fe_3_O_4_ that enables a rapid separation of the immobilized enzyme in a magnetic field. In addition, the Au colloid coating would increase the biocompatibility and immobilization of AChE on GMP, then improve the stability of magnetic Fe_3_O_4_ cores [19,20]. Zirconia nanoparticle (Nano-ZrO_2_) is an inorganic oxide with thermal stability, chemical inertness, and lack of toxicity. Researchers have demonstrated that zirconia has a strong affinity for the phosphoric acid group. It has been used to fabricate various biosensors to determine OP residues [21,22]. Prussian blue is an easily reduced and oxidized material and often used as an electron transfer mediator which can obviously reduce the overpotential of many enzymes such as AChE [25].

In this work, a disposable magnetic composite nanoparticle-modified enzyme biosensor for determination of OPs is described which composed of GMP-AChE/CNTs/ZrO_2_/PB/Nf membrane modified SPCE via an external magnetic field. Based on the inhibition of AChE, the resulting amperometric enzyme biosensor gives good performance in screen determinations of OPs.

## 2. Experimental

### 2.1. Reagents and Apparatus

AChE from *Drosophila melanogaster* (200 units·mg^−1^), acetylthiocholine chloride (ATCh), 1,000 mg·mL^−1^ dimethoate were used as a representative OPs for the inhibition measurements to examine the sensing performance of the enzyme electrode without additional purification; Nafion (0.5% *w*/*v* solution in lower alcohols/water). All above reagents were purchased from Sigma Co. Ltd. CNTs were from Shenzhen Nanotech Port Co. Ltd. (Shenzhen, China). The supporting electrolyte was prepared from 0.1 mol·L^−1^ KNO_3_, and the pH was adjusted with KOH. GMP (Particle Size: 30 nm, concentration: 5 mg·mL^−1^, Shaanxi Lifegen Co. Ltd.). *N*,*N-*Dimethylformamide (DMF), PB and the other regents were of analytical grade and were used as received from Sinopharm Group Chemical Reagent Co. (Shanghai, China). Chinese cabbage samples were from JIAJIALE Supermarket (Ningbo, China). All water used was double-deionized water (Milli-Q, Millipore Corporation).

Cyclic voltammetric (CV) and differential pulse voltammetry (DPV) experiments were performed with a CHI 660B electrochemical workstation (CH Instrument Company, USA). SPCE was purchased from DropSens Corporation (Spain, a carbon electrode served as the working electrode, the auxiliary and reference electrode were carbon electrode and Ag/AgCl electrode respectively). The scanning electron microscope (SEM) images were obtained with a HITACHI S-3400N spectrometer (Hitachi, Japan). The chromatographic analysis was performed on a GC-14B (Shimadzu, Japan); Zetasize Nano ZS90nano particle analyzer (Malvern Instruments Ltd., England); S2 RANGER X-ray fluorescence spectrometer (Bruker, Germany); NdFeB magnet (Hangzhou Magnet Equipment Ltd., China).

### 2.2. Preparation of Zirconia Nanoparticles and GMP-AChE

ZrO_2_ nanoparticles were prepared through a modified sol-gel technique according to the literature [22]. In brief, ZrOCl_2_·8H_2_O (0.75 mol·L^−1^) and 1% PEG 8000 were mixed and added with stoichiometric 0.25 mol·L^−1^ oxalic acid slowly while stirring till a transparent sol of ZrOC_2_O_4_·2H_2_O was produced. After 30 min, 3% oxalic acid (0.25 mol·L^−1^) was added to the sol under stirring. The produced white gelatin was separated from the solution by centrifuge, then washed with ultra purified water, sonicated in anhydrous ethanol, dried in an electric oven, and ground into powder in a mortar. A nanopowder of ZrO_2_ was obtained after calcination at 600 °C for 4 h. The diameters of the ZrO_2_ nanoparticles were characterized by ZEN 3690 analyzer, which indicated a narrow distribution from 18.3 nm to 47.6 nm. The average diameter was 31.5 nm.

The biofunctional AChE-GMP nanocomposite was synthesized according to the literature [19]. 100 μL of GMP solution and 1 mL AChE (1.0 × 10^8^ ng·mL^−1^) were mixed in a centrifuge tube and coupled at 37 °C for 20 min on a 180 r·min^−1^ shaking table to get the GMP-AChE composite magnetic particles. AChE can be readily immobilized on GMP because there are many exposed mercapto groups (−SH) in AChE which can assemble on the surface of nano Au via Au–S covalent bonds [19]. Li [20] also reported that GMP composite can provide a compatible microenvironment for maintaining the activity of the immobilized glucose oxidase which also have many-SH (GOx).

### 2.3. Fabrication of SPCE│CNTs/ZrO_2_/PB/Nf│GMP-AChE

**Figure 1.**
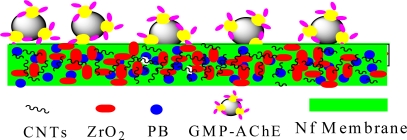
The surface of SPCE│CNTs/ZrO_2_/PB/Nf│GMP-AChE electrode.

1 mL solution containing 0.5 mg·mL^−1^ CNTs, 0.02 mol·L^−1^ PB, 4.0 mg·mL^−1^ ZrO_2_, 0.1% (*w/v*) Nf dissolved by DMF was sonicated for 10 min (enough time to achieve a homogeneous and stable solution). Afterwards, 5 μL of it was dropped on the SPCE surface. The coating was air dried at room temperature for 12 h. Then 1 μL GMP-AChE solution was dropped again and was absorbed by external magnetic field. The enzyme-modified electrode was dried at room temperature and was kept in a refrigerator (at 4 °C) until use. The electrode could be regenerated by simply removing the magnetic field. A schematic diagram of the SPCE│CNTs/ZrO_2_/PB/Nf│GMP-AChE electrode is shown in Figure 1.

### 2.4. The Principle of Inhibition Mechanism and GC Detection

#### 2.4.1. Sample Preparation

Ten grams of Chinese cabbage sample was homogenized in a mortar and extracted for 10 min with 20 mL of acetone. Then, the blend was placed in a closed vial and centrifuged for 5 min at 3,000 rpm. The extraction was repeated twice, and the combined extracts were evaporated by rotary evaporation to 1.0 mL. For sample analysis, blank samples were spiked with dimethoate standards at 1.0, 5.0, 10 ng·mL^−1^.

#### 2.4.2. This Method

**Figure 2.**
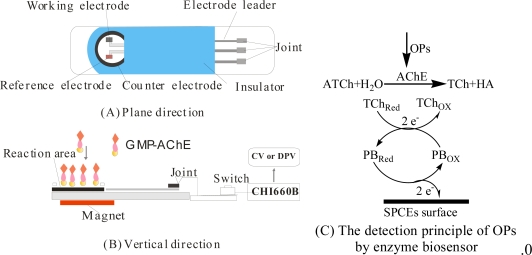
Preview of the enzyme biosensor apparatus from the plane (a), vertical direction (b), and the detection principle of OPs by the biosensor; (c) CV and DPV conditions: 25 μL pH = 7.5 KNO_3_ solution contain 1 mmol·L^−1^ ATCh and the scanning potential range both were +0.6 → −0.2 V.

One of the products of hydrolysis of ATCh with AChE is thiocholine (TCh). Detection of the change of the redox current of TCh (ΔI) can be used to assess the activity of AChE, which can be inhibited by OPs. Thus the change of current of TCh after the biosensor was incubated in OPs solution was linearly correlated with OPs concentration. For OPs determination, the signal was first recorded in 25 μL 0.1 mol·L^−1^KNO_3_ containing 1 mmol·L^−1^ ATCh as substrate. After that, the enzyme biosensor was inhibited for 5 min at 35 °C by 1.0 × 10^−3^, 1.0 × 10^−2^, 1.0 × 10^−1^, 1.0, 10 ng·mL^−1^ dimethoate, respectively. The signal was measured again and the relative decay in the current was calculated in accordance with [8] as a degree of inhibition, *A%* = [(*I*_0_ – *I*)/*I*_0_] × 100%. Where *A* is the degree of inhibition of AChE, There was certain positive correlation between *A* and the concentration of OPs; *I_0_* and *I* are the current values measured prior to and after the enzyme biosensor is treated with an inhibitor. The standard curve method was used for quantitative detection. Since differential pulse voltammetry (DPV) is a sensitive electrochemical method, the *A*% of AChE was investigated using DPV. The preview of amperometric enzyme biosensor apparatus and the detection principle of OPs based on inhibition mechanism are shown in Figure 2.

#### 2.4.3. GC Method [3]

Instrument parameters and operating conditions: flame photometric detector; DB-1701 capillary column (length 3.0 m, i.d. 0.2 mm with 0.25 μm film thickness). The carrier gas was He gas. Sample injection: 3 μL. Column temperature: 240 °C. Detector temperatur: 275 °C. The temperature program was: 80 °C (0.5 min hold) then a 15 °C/min ramp to 240 °C (3 min hold).

## 3. Results and Discussion

### 3.1. Characterization of Different Processes of Modified SPCE

**Figure 3.**
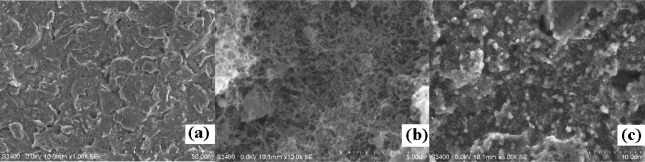
Scanning electron microscope (SEM) images of SPCE (a), SPCE│CNTs/ZrO_2_/PB/Nf (b), SPCE│CNTs/ZrO_2_/PB/Nf│GMP-AChE (c).

Figure 3 displays the SEM images of different preparation stages of the modified electrode. The bare SPCE has an obvious layered structure of graphite (a). A homogeneous, porous, and three-dimensional CNTs film was observed after modification with CNTs/ZrO_2_/PB/Nf-membrane (b). SEM images also showed that there were numerous, well distributed island structures on membrane whose sizes were about 400 nm, much greater than GMP particles. These may be GMP-AChE particles (c).

X-ray fluorescence spectrometry (XRFS) was also used to confirm if the GMP-AChE particles were assembled on the SPCE. The XRFS spectrum of SPCE│CNTs/ZrO_2_/PB/Nf│GMP-AChE electrode showed the characteristic peaks of Zr(*k*α-2.1 keV), Fe (*k*α-6.4 keV), Au(*k*α-9.7 keV) and S (kα-2.3 keV) (not shown here), indicating the surface was modified with GMP-AChE particles.

### 3.2. CVs of the Modified SPCE at Different Processes

**Figure 4.**
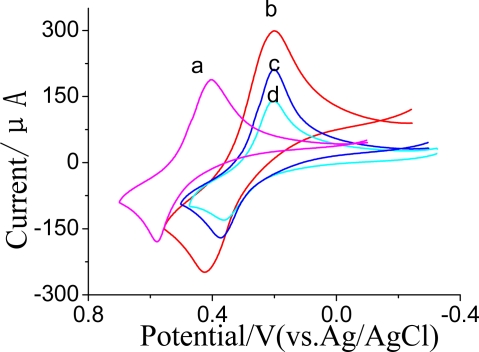
Cyclic voltammetry curves (CVs) of SPCE│CNTs/ZrO_2_/Nf│GMP-AChE (a), SPCE│CNTs/ZrO_2_/PB/Nf│GMP-AChE (b), SPCEs│ZrO_2_/PB/Nf│GMP-AChE (c), and c inhibitted by 1.0 ng·mL^−1^ dimethoate for 5 min at 35 °C (d) in 1.0 mmol·L^−1^ ATCh + 0.1 mol·L^−1^ KNO_3_ (pH = 7.5).

CVs of the SPCE modified with different processes are shown in Figure 4. As expected, the SPCE│CNTs/ZrO_2_/Nf│GMP-AChE electrode (a) incubated in 25 μL 0.1 mol·L^−1^ KNO_3_ containing 1.0 mmol·L^−1^ ATCh as substrate showed an obvious reduction–oxidation peak pair: *E*_pa_ = +0.59 V, *E*_pc_ = + 0.41V, corresponding to the redox reactions of TCh, the products of hydrolysis of ATCh with AChE. There was an increase in overall voltammetric signal after the Nf-membrane was added with PB (SPCE│CNTs/ZrO_2_/PB/Nf│GMP-AChE electrode, (b). The modified electrode reduces the working potential and increases the reversibility of electrode reactions to provide higher sensitivity and selectivity of the signal [25]. It could be expressed with this equation (where PB_Red_ and PB_Ox_ are the reduced and oxidized forms of the mediator):

$$\begin{array}{c} \underset{\left(\text{ATCh}\right)}{{\left({\text{CH}}_{3}\right)}_{3}N^{+}{\text{CH}}_{2}{\text{CH}}_{2}\text{SC}(O){\text{CH}}_{3}}\;\overset{\text{AChE}+H_{2}O}{\to }\;\underset{\left(\text{TCh}\right)}{{\left({\text{CH}}_{3}\right)}_{3}N^{+}{\text{CH}}_{2}{\text{CH}}_{2}\text{SH}}+\underset{\left(\text{HA}\right)}{{\text{CH}}_{3}\text{COOH}}\\ 2{\left({\text{CH}}_{3}\right)}_{3}N^{+}{\text{CH}}_{2}{\text{CH}}_{2}\text{SH}+2{\text{PB}}_{\text{OX}}\to {\left({\text{CH}}_{3}\right)}_{3}N+{\text{CH}}_{2}{\text{CH}}_{2}{\text{SSCH}}_{2}{\text{CH}}_{2}N^{+}{\left({\text{CH}}_{3}\right)}_{3}+2{\text{PB}}_{\text{Red}}\\ {\text{PB}}_{\text{Red}}-2e^{-}⇌{\text{PB}}_{\text{OX}} \end{array}$$

We also found there was an obvious increase in overall voltammetric signal before (c) and after (b) CNTs immobilized in the Nf-membrane, which was due to the fact CNTs can promote the electron transfer of PB significantly. The current of the SPCE│CNTs/ZrO_2_/PB/Nf│GMP-AChE electrode (b) decreased obviously after it was incubated in 1.0 ng·mL^−1^ dimethoate for 5 min at 35 °C (d); this was due to the activity of AChE being inhibited by the OPs. The digressive current value was proportional to the OPs concentration.

### 3.3. Optimization of the Preparation and Analysis Conditions

#### 3.3.1. Concentration of CNTs, ZrO_2_ and PB in Nf-membrane

**Figure 5.**
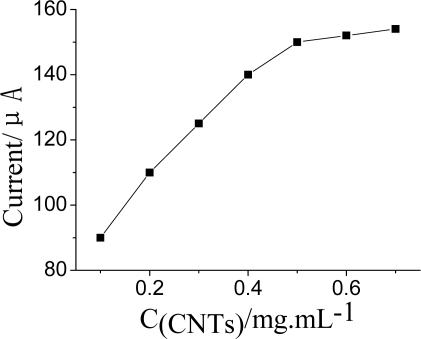
Effect of the concentration of CNTs in Nf membrane on DPV current. DPV conditions: 25 μL pH = 7.5 KNO_3_ solution contain 1.0 mmol·L^−1^ ATCh and after the AChE was inhibited by OPs for 5 min at 35 °C.

The effect of CNTs concentration on current response was studied between 0.0 and 0.8 mg·mL^−1^. The anodic current gradually increased with increasing CNTs content and reached a maximum at 0.5 mg·mL^−1^, as shown in Figure 5. Further increasing the amount of CNTs lead to a constant value, possibly because of increased resistance and double layer capacitance of the modified electrode, so 0.5 mg·mL^−1^ CNTs was used for preparation of the biosensor.

**Figure 6.**
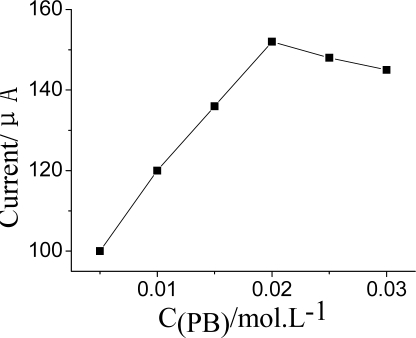
Effect of the concentration of PB in Nf membrane on DPV current. DPV conditions: 25 μL pH = 7.5 KNO_3_ solution contain 1.0 mmol·L^−1^ ATCh and after the AChE was inhibited by OPs for 5 min at 35 °C.

The influence of PB concentration on current response by the sensor has been also studied (Figure 6). The DPV peak current increased when the concentrion of PB was enhanced in the Nf-membrane. However, when it exceeded 0.02 mol·L^−1^, the DPV peak current tended to decline. It was presumed that when the amount of PB is too much it would easily leak away from the Nf membrane from the electrode surface into the electrolyte. Especially for those soluble mediators with low molecular weight, such as ferrocene (Fc) [19] or PB [25]. This resulting in poor sensor response performance. Therefore, the concentration of PB was fixed at 0.02 mol·L^−1^ for preparation of the Nf membrane.

**Figure 7.**
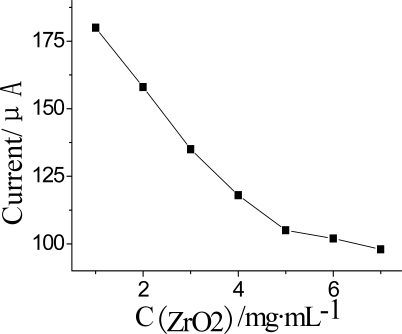
Effect of the concentration of ZrO_2_ in Nf membrane on DPV current. DPV conditions: 25 μL pH = 7.5 KNO_3_ solution containing 1.0 mmol·L^−1^ ATCh and after the AChE was inhibited by OPs for 5 min at 35 °C.

The effect of content of ZrO_2_ in the Nf-membrane is shown in Figure 7. The cathodic DPV peak current decreased with ZrO_2_ after the AChE was inhibited by OPs for 5 min at 35 °C. Therefore, an optimal ZrO_2_ concentration of 4.0 mg·mL^−1^ was chosen to fabricate biosensor to best satisfy the sensitivity demands.

#### 3.3.2. The Amount of Enzyme Immobilized on Electrode Surface

**Figure 8.**
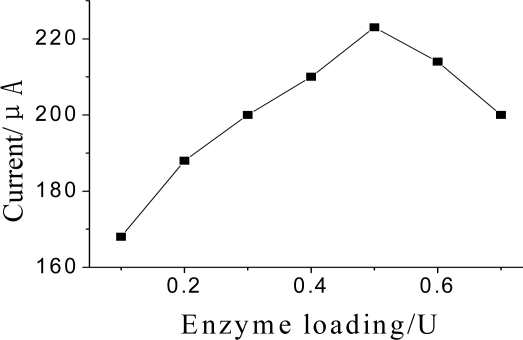
Effect of the amount of GMP-AChE absorbed on DPV current. DPV conditions: 25 μL pH = 7.5 KNO_3_ contain 1.0 mmol·L^−1^ ATCh.

The amount of enzyme immobilized on electrode surface was another important aspect of the preparation. Figure 8 displays the effect of enzyme loading on amperometric response. When the volume of employed GMP-AChE was increased, the current of biosensor increased and then lead to a decrease of the response. More than 0.5 U adsorbed enzyme was not sufficiently stable, owing to limited electrode area, indicating saturation of enzyme loading. Therefore, the amount of enzyme immobilized on the electrode surface was set at 0.5 U.

#### 3.3.3. Effect of pH, Temperature, Inhibition Time on Current Response

**Figure 9.**
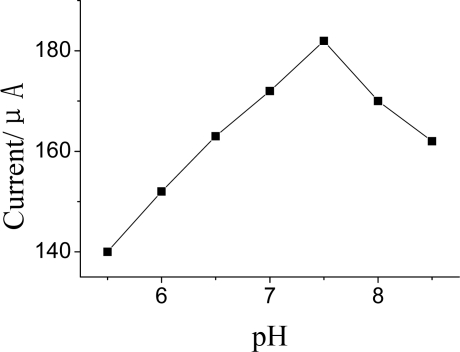
Effects of pH on DPV current DPV conditions: 25 μL pH = 7.5 KNO_3_ contain 1.0 mmol·L^−1^ ATCh.

The bioactivity of the immobilized AChE depended on solution pH. Figure 9 shows the relationship between catalytic peak current of the response of AChE to ATCh and solution pH. Obviously, the maximum peak current was obtained at pH 7.5 in the pH range from 5.5 to 8.5. This result was close to that reported for the soluble enzyme, indicating that GMP-AChE composite did not alter the optimum pH for catalytic behavior of the enzyme. Therefore, pH 7.5 was used in the detection.

**Figure 10.**
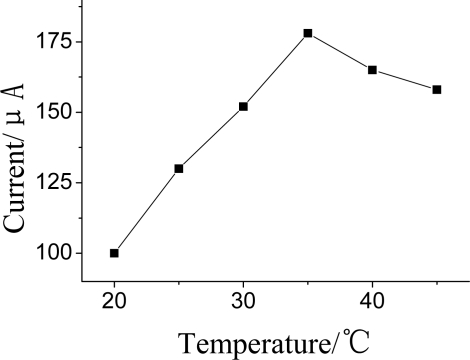
Effects of incubation temperature on DPV current. DPV conditions: 25 μL pH = 7.5 KNO_3_ contain 1.0 mmol·L^−1^ ATCh.

The effect of temperature on the DPV current was examined at the range from 20 to 45 °C (Figure 10). It was found that the current response increased with the increasing temperature up to 35 °C. However, it could not cause significantly change when temperature was changed over 35 °C Thus, 35 °C was selected as the optimum incubation temperature.

**Figure 11.**
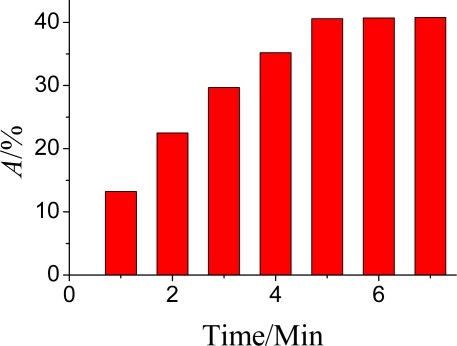
Effects of inhibition time on *A*%. DPV conditions: 25 μL pH = 7.5 KNO_3_ containing 1.0 mmol·L^−1^ ATCh and after the AChE was inhibited by OPs at 35 °C.

The influence of the inhibition time on *A*% was also investigated (Figure 11). In the incubating solution, It took some time for the OPs to complete inhibit the bioactivity of the immobilized AChE. The inhibition times were 1, 2, 3, 4, 5, 6 and 7 min, using the same analyte concentrations in this study. The *A*% obtained in this study increased with the inhibition time rapidly up to 5 min and after that the variation slowed. Therefore, the inhibition time of 5 min was adopted in the subsequent work.

### 3.4. The Detection Character of OPs

**Figure 12.**
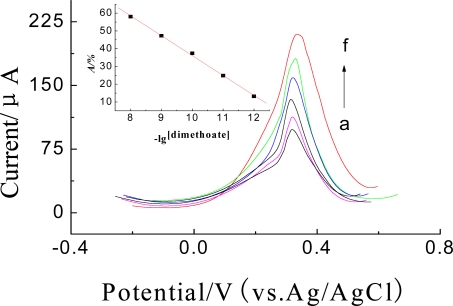
DPVs of the enymze biosensor inhibitted by (a) 10, (b) 1.0, (c) 0.1, (d) 1.0 × 10^−2^, (e) 1.0 × 10^−3^, (f) 0 ng·mL^−1^ dimethoate and parathion for 5 min in 25μL pH = 7.5 KNO_3_ solution at 35 °C. Inset: The calibration curve of *A*% *vs.* dimethoate.

Under the optimal conditions mentioned above, *A*% of the enzyme biosensor was related linearly to the concentration of dimethoate in the range from 1.0 × 10^−3^ to 10 ng·mL^−1^ with a detection limit of 5.6 × 10^−4^ ng·mL^−1^ (S/N = 3) (as seen from Figure 12). The regression equation is: *A*% = 148.13–11.20c (*R*^2^ = 0.9983). It indicated that the biosensor was more sensitive in comparison with the values reported in the literature [5] by HPLC and by voltammetry [16] but without a nanoparticle modified enzyme electrode. After an overall determination, the modified electrode can be regenerated by removing the magnetic field. Moreover, the proposed biosensor is superior to that without ZrO_2_ in the Nf membrane (Linear range: 5.0 × 10^−2^–10 ng·mL^−1^, detection limit: 2.0 × 10^−2^ ng·mL^−1^). The comparison of this method with other enzyme biosensors was shown in Table 1.

### 3.5. Repeatability, Stability and Reproducibility

One, five and ten ng·mL^−1^ dimethoate solution was detected five times with the same modified SPCE regenerated after every determination. As a result, the relative standard derivation (R.S.D.) for the DPV cathodic peak current were 3.2%, 3.5% and 2.9% (n = 5). Additionally, a 1.0 mmol·L^−1^ substrate ATCh solution inhibited by 5.0 ng·mL^−1^ dimethoate at 35 °C for 5 min was detected with five modified SPCEs prepared by the same procedure, and the R.S.D. for the DPV cathodic peak current was 5.2% (n = 4).

The stability of the enzyme biosensor was investigated, and it’s response current reached 93.0% and 88.0% of its initial current to 1.0 mmol·L^−1^ ATCh, respectively, after storage at 4 °C for 30 and 40 days. The good stability may due to the fact that the stability of GMP was consistent and protein molecules were attached firmly onto the GMP surface.

After every determination, the modified electrode can be regenerated by removing the magnetic field and washing with double-deionized water, 5 μL GMP-AChE composite magnetic particulate was absorbed on the surface again through magnet. The response current reached 97.0% of its initial current to 1.0 mmol·L^−1^ ATCh, indicating that the biosensor has good reproducibility.

### 3.6. Determination of Dimethoate in Chinese Cabbage

Prior to determination, the Chinese cabbage samples were processed according to 2.4.1. Then 2 μL of the extracted solution was dissolved in 25 μL KNO_3_ solution of pH 7.5 and was dropped on SPCE for determination. A standard addition method was adopted to assess the reliability. Samples were also analysed by GC methods. The recovery rates in Chinese cabbage exhibited a range of 88.0% to 105% and the results are shown in Table 2.

## 4. Conclusions

In this study, Fe_3_O_4_/Au and ZrO_2_ NPs composite particles had been successfully employed as sensitive membrane matrix to immobilize AChE enzyme on a SPCE’ surface to fabricate a novel disposable OPs biosensor. The results showed that the sensor could rapidly, sensitively and specifically screen/determine OPs under optimal conditions. The biosensor has such attractive advantages as: (1) the biosensor exhibited a fast response, excellent linear detection range and high sensitivity to OPs due to the conductive Fe_3_O_4_/Au NPs (GMP) being used to provide a large electrode surface area to amplify the current response signal of TCh and enhance its sensitivity. Furthermore, its manufacture cost is lower than common enzyme electrodes because GMP is an easier enzyme immobilization mediator which simplifies the experimental design and reduces the consumption of expensive reagent; (2) the biosensor has specific adsorption to OPs, because ZrO_2_ NPs have specificity to OPs which can remarkably decrease the interference of other matters to inhibit the activity of AChE; (3) the biosensor’s surface can be renewed easily due to the easy removal of Fe_3_O_4_/Au/AChE from the biosensor surface by applying an external magnetic field thanks to its super-paramagnetism. Thus, the proposed biosensor can be a useful approach to improve the performance of the AChE-inhibition based biosensors and an alternative method to other conventional assays for screening and determination of OP residues in vegetables and other samples.

## Figures and Tables

**Table 1.** The comparison of this method with other enzyme biosensors.

**Method [ref.]**	**Linear range/ng·mL^−1^**	**Detection limit/ng·mL^−1^**	**Detection time/min**	**Stability time/d**
This method	1.0 × 10^−3^–10	5.6 × 10^−4^	10	40
AChE/Au//PB/GCE [8]	5.0 × 10^−2^–50	2.0 × 10^−2^	15	19
AChE/Nf/TCNQ/SPCE [9]	1.0 × 10^2^–1.0 × 10^4^	2.1	12	40
AChE/Au-Fe_3_O_4_/GCE [10]	1.7 × 10^−1^–2.2 × 10^2^	8.6 × 10^−2^	15	35

**Table 2.** Determination results of dimethoate in Chinese cabbage samples (n = 3, ng·mL^−1^).

**Sample**	**Number**	**This method**	**Add**	**Found**	**GC Found**	**Recovery/%**
A	1	0	1.0	8.8 × 10^−1^	9.2 × 10^−1^	88.0
	2	0	5.0	4.7	4.8	94.0
	3	0	10	9.8	9.9	98.0
B	1	0	1.0	9.7	9.9	97.0
	2	0	5.0	5.2	5.1	104.0
	3	0	10	10.5	10.3	105.0
C	1	2.5	2.5	5.1	5.1	104.0
D	1	1.5	2.5	3.9	4.0	93.3
